# Investigation of the Food-Transmitted Parasites *Trichinella* spp. and *Alaria* spp. in Wild Boars in Greece by Classical and Molecular Methods and Development of a Novel Real-Time PCR for *Alaria* spp. Detection

**DOI:** 10.3390/ani11102803

**Published:** 2021-09-26

**Authors:** Dimitris Dimzas, Taxiarchis Chassalevris, Zanda Ozolina, Chrysostomos I. Dovas, Anastasia Diakou

**Affiliations:** 1 Laboratory of Parasitology and Parasitic Diseases, School of Veterinary Medicine, Faculty of Health Sciences, Aristotle University of Thessaloniki, 54124 Thessaloniki, Greece; dimzas@vet.auth.gr; 2 Diagnostic Laboratory, School of Veterinary Medicine, Faculty of Health Sciences, Aristotle University of Thessaloniki, 11 Stavrou Voutyra Str., 54627 Thessaloniki, Greece; taxiarchis@hotmail.com (T.C.); dovas@vet.auth.gr (C.I.D.); 3 Institute of Food Safety, Animal Health and Environment “BIOR”, Lejupes Str. 3, 1076 Riga, Latvia; zanda.ozolina@bior.lv

**Keywords:** *Alaria alata*, *Trichinella*, wild boar meat, Greece, real-time PCR

## Abstract

**Simple Summary:**

There are many parasites that may be transmitted to humans via food, and meat is a major source of such infections. *Trichinella* spp. is one of the most important meat-transmitted parasites, while *Alaria* spp. may be considered an emerging pathogen, albeit to date rarely reported in humans. Raw and undercooked wild boar meat has been proven as a major source of human infection by both parasites. In the present study, an investigation of the presence of these parasites in wild boar meat was conducted for the first time in Greece. Classical parasitological methods and molecular techniques were implemented for the examination of samples collected from 128 hunted wild boars, and none of them were found positive for *Trichinella* spp. or *Alaria* spp. For the detection of *Alaria* spp., a novel molecular method was developed, offering a powerful complementary diagnostic tool that may be useful for the epizootiological surveillance of the parasite. The epizootiology/epidemiology, clinical implications, and importance of monitoring of these parasitic infections are briefly discussed.

**Abstract:**

Foodborne parasitic diseases represent a major threat to public health. Trichinellosis, caused by the nematode parasite *Trichinella* spp., is one of the most important foodborne diseases, while alariosis, caused by the trematode parasite *Alaria* spp., is less common in humans, and rare cases have been reported only in the USA and Canada. Both parasites can infect humans via the consumption of raw or undercooked wild boar meat. In order to investigate the prevalence of these parasites in wild boar meat in Greece, samples from the diaphragm pillars and the region of the mandibular angle from 128 wild boars, hunted in Greece, were collected. The samples were examined by classical parasitological (compression, artificial digestion, and *Alaria* spp. migration) and by molecular (real-time PCR) methods. For *Trichinella* spp. an existent real-time PCR detecting all species likely to be present in Greece was applied, while for *Alaria* spp. a real-time PCR was developed, employing an LNA TaqMan probe targeting the large subunit ribosomal RNA gene. All examined wild boar samples from Greece resulted negative for *Trichinella* and *Alaria* species, indicating a low prevalence of infection in the examined population. The novel real-time PCR for *Alaria* spp. has 81.5% amplification efficiency and is able to detect 0.12 larvae per 50 g of tissue and could be utilized as a complementary to AMT diagnostic tool in surveillance.

## 1. Introduction

Foodborne diseases (FBDs) are an important public health problem worldwide, with economic and social implications that involve a wide range of pathogens, i.e., viruses, bacteria, and parasites [1,2]. Foodborne parasitic diseases (FBPDs), many of which are developed after the consumption of infected meat (e.g., toxoplasmosis, sarcocystosis, taeniosis, cysticercosis), represent a major part of FBDs but have not been adequately studied or monitored due to their complex epidemiology/epizootiology and their usually non-acute but rather chronic clinical implications [3,4]. Among food transmitted parasites, *Trichinella* spp., the agent of trichinellosis, and *Alaria* spp., the agent of alariosis, are of particular interest due to their potentially severe or even fatal outcome in human infections. Trichinellosis and alariosis share some common features in terms of transmission, diagnosis, and prevention. For example, they are exclusively foodborne, transmitted by the consumption of raw or undercooked infected meat, and domestic pig and wild boar meat are a common source of human infection. Consequently, suitable food inspection methods and thermal treatment of meat may prevent human infection by both parasites [5,6].

*Trichinella* spp. are nematode parasites, affecting mainly mammals but also birds and some reptile species. Their life cycle is completed in the same host: adult parasites inhabit the intestine, and females lay larvae in the intestinal mucosa. These larvae migrate via the lymphatic and blood vessels to the muscles, especially the highly oxygenated ones, which represent their predilection sites. A new host is infected by consuming raw or undercooked, infected meat. The genus *Trichinella* is divided into two clades (encapsulated or non-encapsulated), depending on the presence or absence of a capsule, enclosing the parasite in the hosting muscle cell (“nurse cell”) [7]. The encapsulated species are *Trichinella spiralis*, *Trichinella britovi*, *Trichinella nativa Trichinella murrelli, Trichinella nelsoni, Trichinella patagoniensis, Trichinella chanchalensis,* and the genotypes T6, T8, and T9, while the non-encapsulated species are *Trichinella pseudospiralis, Trichinella papuae,* and *Trichinella zimbabwensis* [8]. Humans are susceptible to different *Trichinella* species; however, *T. spiralis* is the most commonly identified in human cases [7]. The World Health Organization (WHO) ranks trichinellosis as one of the most important FBD due to its impact on human health [3,9].

The trematode *Alaria* spp. has an indirect, three-host life cycle. The adult parasite inhabits the intestine of carnivores of the families Canidae, Felidae, and Mustelidae (definitive hosts), and the larval stages develop in water snails (first intermediate host) and amphibians (second intermediate host), where the parasite develops into mesocercariae, also known as *Distomum musculorum suis* (DMS), which is the infective stage for definitive hosts. Mesocercariae are an interjectional stage and change inside the definitive host to metacercariae and then to adult parasites [6]. Paratenic hosts play an important role in the life cycle of *Alaria* spp. by maintaining and accumulating mesocercariae in their tissues, spreading the infection in more animal species, thus facilitating the infection of the definitive hosts [6]. The migration of the mesocercarial stage is the cause of alariosis that affects both humans and animals. Humans become infected by eating raw or undercooked meat from infected second intermediate and paratenic hosts, such as wild boars [6]. The species *Alaria americana* is incriminated for most human cases, and it is present in the Americas where the species *Alaria mustelae*, *Alaria intermedia*, *Alaria marcianae*, *Alaria arisaemoides*, *Alaria canis*, and *Alaria taxideae* have also been identified [6,10,11]. *Alaria alata* is the species found in Europe, and it is closely related to the zoonotic *A. americana*, as it has a similar life cycle with various wild animals as paratenic hosts that harbor mesocercariae. Furthermore, it has been shown that *A. alata* mesocercariae are infectious for primates (the Rhesus monkey *Macaca mulatta*) [12], and thus, it is most likely infectious for humans as well. As a possible cause of FBPD, *A. alata* is included in the list of zoonotic agents in some countries, e.g., Switzerland and Germany [6,11].

In Greece, human trichinellosis was reported for the first time in 1946 [13]. Since then, the infection has been sporadically reported in humans and animals [14]. Epizootiological studies on trichinellosis in Greece are limited [15,16], leaving a significant gap in our knowledge on the frequency of parasitism in animals, as well as the species of parasites that are enzootic in the country. On the other hand, *Alaria* spp. has been reported as an adult in several species of wild and domestic carnivores [17,18,19,20] but never in the form of DMS in meat or other tissue.

Both *Trichinella* spp. and *Alaria* spp. can be detected in meat by classical parasitological methods, i.e., tissue compression and artificial digestion (AD), while *Alaria* spp. can be also detected by *Alaria* spp. migration technique (AMT) [11]. At the food inspection level and according to the EU legislation [21], examination for *Trichinella* is obligatory for all slaughtered pigs and wild boars, as well as for meat from other game animals, and it is performed by magnetic stirrer artificial digestion before releasing to the consumption. Molecular methods have been developed mainly for the detection and identification of *Trichinella* spp. at the experimental/investigational level [22,23,24], while for the molecular detection of *Alaria* spp. only one conventional PCR method has been published so far [25].

The development of novel, sensitive, fast, and cheap methods for the detection of these important food-transmitted parasites should always be a priority for the scientific community. This is particularly true for alariosis, as it is considered an emerging and potentially severe disease for humans [6,26], and to date, no such modern methods have been developed.

In this context, the aims of the present study were (a) to evaluate the prevalence of *Trichinella* spp. and *A. alata* in wild boars (*Sus scrofa*) in Greece, by classical parasitological and by molecular methods, and (b) to develop and apply a highly sensitive and specific real-time PCR protocol for the detection of *A. alata* in tissues.

## 2. Materials and Methods

### 2.1. Samples

#### 2.1.1. Field Samples

**Figure 1:**
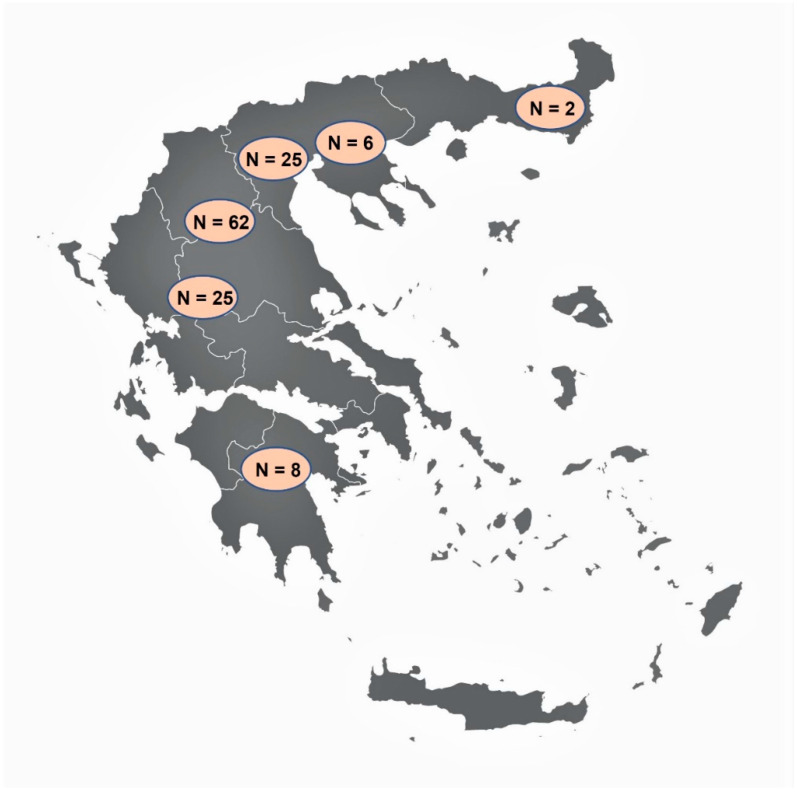
The map of Greece showing the areas where the examined wild boars originated from, and the number of animals (N) examined from each area (vector map source: https://freevectormaps.com/greece/GR-EPS-01-0002?ref=atr, accessed on 21 September 2021).

Tissue samples from 128 wild boars were collected during the hunting season 2019–2020 (i.e., October 2019 to January 2020). Sampling was performed voluntarily by hunters, mostly in areas of northern Greece (Figure 1), after a brief training regarding the procedure. For *Trichinella* spp. detection, a minimum of 15 g muscle tissue from the pillars of the diaphragm (diaphragm pillars, DP) was collected, as this is one of the predilection sites of the parasite [27]. For *Alaria* spp. detection, a minimum of 110 g of tissue from the region of the mandibular angle including, connective, fat, glandular, lymphatic, and muscle tissues (mandibular area tissues, MT) were sampled, as it has been proven that this is one of the best sites for *Alaria* spp. detection [28]. All samples were stored in separate containers at 4–7 °C and sent to the Laboratory of Parasitology and Parasitic Diseases, School of Veterinary Medicine, Aristotle University of Thessaloniki, at the latest 48 h after collection.

#### 2.1.2. Control Samples

*Trichinella* spp. of all species that are present or could be present in Greece, i.e., *T. spiralis*, *T. britovi*, and *T. pseudospiralis*, conserved in 90–95% ethyl alcohol, were kindly provided by Dr. Edoardo Pozio, Istituto Superiore di Sanità, Rome, and were used as reference DNA and positive controls.

*Alaria alata* parasites originated from the collection of the Institute of Food Safety, Animal Health and Environment “BIOR”, Latvia. More precisely, 25 mesocercariae isolated by AMT from wild boar muscle tissue were used for reference DNA. Furthermore, six *A. alata* infected wild boar meat samples from Latvia were used as positive controls. The parasitic load in these six control samples was determined by AMT and it was 7 (in 3 of the samples), 1, 3, and 76 mesocercariae per 50 g of meat tissue.

### 2.2. Examination Methods

#### 2.2.1. Tissue Compression

From each animal, tissue samples of 1g each from DP and MT were examined by the tissue compression method [21,29]. Briefly, the samples were cut to smaller pieces (rice grain size), compressed between two glass plates (compressorium), and examined under the stereomicroscope (20–40× magnification) and the optical microscope (100× magnification).

#### 2.2.2. Processing of DP Samples and Magnetic Stirrer Artificial Digestion

All the DP tissue samples were examined by the magnetic stirrer AD performed according to the standard protocol and the latest directives of the European Union for the control of trichinellosis [21,30,31]. Initially, 11 g of muscle tissue were minced with 11 mL of saline (NaCl 0.9%) in a meat mincer at max speed for 2–3 s, then the blender was opened, and any muscle piece at the walls of the bowl was transferred to the bottom and blending continued with 2–3 s bursts until no visible pieces of meat remained [30]. An aliquot of 1 mL of the homogenized material was stored at −80 °C, prior to processing for DNA extraction and further analysis by PCR. The rest of the material was mixed with the artificial digestion fluid [1.6 mL 25% HCl and 1 ± 0.02 g of pepsin (1:10,000 NF, 1:12,500 BP, 2000 FIP) to 200 mL of tap water at 46–48 °C]. The fluid was stirred vigorously for less than 60 min to ensure adequate digestion. Once the digestion was performed, the digestion fluid was poured through a 180 µm sieve into a sedimentation glass funnel. After 30 min, the supernatant was discarded, and at least 80 mL of the sediment were examined under a stereomicroscope at 20–40× magnification.

#### 2.2.3. Processing of MT Samples and *Alaria* spp. Migration Technique

All the MT samples were examined by AMT according to the classical protocol of larval migration [32], implementing the modifications proposed by Riehn et al. [28]. In brief, 50 g were chopped into small pieces (approximately 0.5 cm edge length), transferred to a sieve, and immersed over a glass funnel filled with warm tap water (46–48 °C, cooling to room temperature) for 90–120 min. At least 40 mL of the sediment were examined under a stereomicroscope at 20–40× magnification.

For DNA extraction, an additional quantity of 50 g of tissues, were minced with 25 mL of PBS to ensure complete homogenization. An aliquot of 1 mL of the homogenized material was stored at −80 °C, prior to processing for DNA extraction and further analysis by PCR.

#### 2.2.4. Molecular Based Methods

All DP and all MT samples (a total of 256 samples) were examined separately for *Trichinella* spp. and for *Alaria* spp. detection, i.e., each animal was examined by molecular-based methods twice for each parasite (DP and MT).

DNA was extracted from the tissue samples according to a previously described protocol [33], with some modifications. Specifically, 450 μL of the DP and MT homogenized material was transferred to 1950 μL of lysis buffer [4.2 M guanidinium iso-thiocyanate, 1.2 Μ guanidinium hydrochloride, 50 mM Tris-HCl (pH 7.5), 25 mM EDTA, 1% N-lauroylsarcosine, 2% Triton X-100, pH 7.0] and vortexed thoroughly. Then, 250 μL of lysate was transferred to a 2 mL microcentrifuge tube and mixed with 850 μL of lysis buffer, followed by the addition of 600 μL phenol (equilibrated, stabilized, pH 7.8–8.2) and 200 μL of chloroform–isoamyl alcohol (24:1). The remaining steps of the protocol were performed as described previously [33], and 60 μL of preheated (75 °C) elution buffer (5 mM Tris-HCl, pH 8.5) was used for DNA recovery.

DNA purification from ethyl alcohol stored reference larvae was also performed using the aforementioned published protocol [33], with some modifications. Specifically, following centrifugation (500× *g*, 10 min), the supernatant was discarded, and the resulting pellets were resuspended in 250 μL of phosphate-buffered saline (PBS). The pellet suspensions (250 μL) were mixed with 850 μL of Lysis buffer. The remaining steps of the protocol were performed as described previously (2)l and 60 μL of preheated (75 °C) elution buffer (5 mM Tris-HCl, pH 8.5) was used for DNA recovery.

For the detection of *Trichinella* spp. a real-time PCR was performed according to a published protocol [24] able to detect all known *Trichinella* species in muscle samples from various wild animals.

For the molecular detection of *A. alata* DNA, a real-time PCR methodology was developed, employing an LNA TaqMan probe and targeting the large subunit ribosomal RNA gene (lsrDNA), as previously suggested for a respective conventional PCR protocol [25]. The oligonucleotide selection was based on homologous lsrDNA gene regions of *A. alata*, *A. americana*, *A. marcianae*, and *A. mustelae* (N = 27) and closely related *Diplostomum* spp. (N = 14) available in GenBank. All sequences were imported in MEGA 7 software [34] and were aligned by MUSCLE [35]. Primers and probes for the specific detection of all *Alaria* species were designed from conserved genomic regions and were further evaluated for their melting temperature (*Tm*) and self- or hetero- dimer formation using the IDT OligoAnalyzer 3.1 software (https://eu.idtdna.com/calc/analyzer, accessed on 15 June 2021) [36]. Selected primers AlarDME1F2 and AlarDME1R2 amplify a 309 bp region (Table 1).

A highly conserved short region located 115 nucleotides downstream of the AlarDME1F2 3′-end was identified as suitable for the design of a probe containing locked nucleic acid (LNA) modifications. For efficient mismatch discrimination with the closely related *Diplostomum* spp. sequences presenting two specific A/G mutations in the homologous targeted probe region, a triplet of LNA modifications with the central base of the triplet at each mismatch site, was incorporated [37,38].

The optimized real-time PCR amplification was performed in a final volume of 20 μL. Reactions comprised 1 × PCR buffer [(Tris·Cl, KCl, (NH_4_)2SO_4_, 15 mM MgCl_2_; pH 8.7 (20 °C), (Qiagen, Hilden, Germany)] and 5 U of HotStarTaq DNA polymerase (Qiagen, Hilden, Germany), 3 mM MgCl_2_, 0.2 mM of each dNTP, the primers AlarDME1F2 and AlarDME1R2 (Integrated DNA Technologies; IDT, Coralville, IA, USA), the LNA TaqMan probe AlAlprobe (IDT) at concentrations shown in Table 1, nuclease-free water, and 2 μL of DNA extract. The assay was performed using a CFX96 Touch™ Real-Time PCR Detection System (Bio-Rad Laboratories, Hercules, CA, USA), with the cycling conditions shown in Table 1. Fluorescence was measured at the end of each cycle and analysis of fluorescence data was performed using CFX Maestro Software (v4.1, Bio-Rad Laboratories).

The PCR efficiency for *Alaria* spp. detection was determined by amplification of 10-fold serial dilutions of pure *A. alata* genomic DNA prepared in siliconized tubes with nuclease-free water containing 50 ng of carrier RNA (QIAGEN, Hilden, Germany) per µL and were stored at −80 °C. Dilution series contained 2.5 × 10^−2^–2.5 × 10^−6^ larvae per PCR assay in 4 replicates. Specificity was assessed by testing purified genomic DNA derived from negative samples spiked with *Trichinella spiralis*, *Trichinella pseudospiralis,* and *Trichinella britovi* genomic DNA. In order to evaluate the presence of PCR inhibitors in muscle samples, PCR assays of genomic DNA of AMT negative muscle tissue (n = 3) were spiked with *A. alata* genomic DNA (2.5 × 10^−2^ larvae) and was tested in parallel with *A. alata* genomic DNA samples (2.5 × 10^−2^ larvae per PCR assay).

## 3. Results

### 3.1. Field Samples’ Results

All classical parasitological methods, i.e., compression, AD, and AMT, of all examined samples were negative for *Trichinella* spp. larvae and for *Alaria* spp. mesocercariae. The results of the classical parasitological methods were confirmed by the molecular methods as real-time PCRs of all DP and MT were negative for *Trichinella* spp. and *Alaria* spp. infection.

### 3.2. Performance of Real-Time PCR for Alaria *spp.* Detection

**Figure 2:**
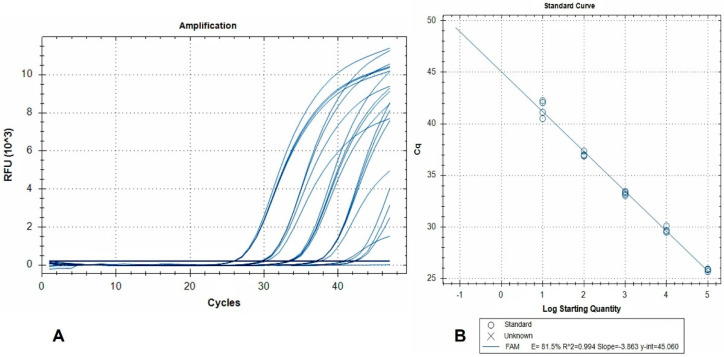
(**A**) Amplification plots (FAM fluorescence signals) generated by a dilution series of pure *A. alata* genomic DNA. Curves represent 10-fold DNA serial dilutions, from 2.5 × 10^−2^ (left) to 2.5 × 10^−6^ (right) larvae per reaction, tested in 4 replicates. Non-template controls are also included; (**B**) the corresponding standard curve. RFU: relative fluorescence units.

The standard curve and linear regression analysis revealed a linear range of quantification extending over a 5-log10 range (2.5 × 10^−2^–2.5 × 10^−6^ larvae per reaction), and amplification efficiency of 81.5% (Figure 2).

This indicates that detection of 2.5 × 10^−6^ larvae per reaction is achievable, and when accounting for the dilutions due to DNA extraction from 50 g AMF samples, this level of sensitivity corresponds to 0.12 larvae per 50 g.

The developed real-time PCR assay successfully tested positive all naturally infected tissue samples (i.e., positive controls). In contrast, the assay delivered a negative result when testing purified genomic DNA derived from negative samples spiked with *T. spiralis*, *T. pseudospiralis,* and *T. britovi* genomic DNA. The observed *Ct* shift (0.073) between the *A. alata* spiked DNA extracts and inhibition controls was negligible, indicating no inhibition of the real-time PCR.

## 4. Discussion

Food transmitted parasites and related diseases are often neglected, at the level of food safety control systems and etiological diagnosis. The apparent reasons are that infected animals usually do not show any clinical signs, associated monetary losses are not easy to determine, and FBPDs often remain subclinical in humans [39]. However, occasionally, FBPDs have significant health implications and may be fatal.

Trichinellosis is transmitted to humans by the consumption of raw or inadequately cooked meat and raw cured meat products. Humans trichinellosis may remain asymptomatic; however, clinical manifestations often include an initial phase of nonspecific symptoms, e.g., headache, malaise, fever, and gastrointestinal disorders (epigastric pain, diarrhea, nausea, and vomiting) and a subsequent phase characterized by myalgia, arthralgia, dyspnoea, as well as some characteristic, but not always present, lesions, i.e., periorbital or facial edema and subungual petechiae. Severe complications such as myocarditis and encephalitis can also occur and may lead to death [7,40]. Despite the reduction of cases numbers in the past 10 years, human trichinellosis is still reported in Europe. During the period 2013–2017, 1022 new cases have been reported in the EU [41], while according to the latest report, 96 cases of human trichinellosis were confirmed in 2019, i.e., 0.02 cases per 100,000 population [42].

On the other hand, to date, human alariosis has not been confirmed in Europe [43]. In their review, Möhl et al. [6] cite seven human cases, as attributed to *Alaria* spp., albeit the etiological diagnosis was not always confirmed. All these cases were reported between 1969 and 1993 in the USA and Canada and in those for which the investigation was fruitful, indicated that the source of human infection was game meat (paratenic host) or frog legs (second intermediate host). Ocular (unilateral decreased vision, diffuse unilateral subacute neuroretinitis), skin (intradermal swellings), and generalized parasitism have been described in human alariosis [44,45,46,47]. The case of generalized infection had a fatal outcome, as thousands of mesocercariae migrated to vital organs of the patient, who died just 9 days from the onset of symptoms, due to respiratory insufficiency caused by extensive pulmonary hemorrhage [45]. The various paratenic hosts of the parasite that include wild mammals and birds may have an important role in the transmission of this zoonosis, especially given the growing popularity and need for game and organic meat [10].

*Trichinella* spp. and *Alaria* spp. have been found in wild boar meat (in single and mixed infection), and therefore, this kind of meat has been reasonably characterized as a potentially important source for human infection by both parasites [6]. According to a quantitative microbial risk assessment applied by Franssen et al. [5], wild boar meat is incriminated for 55% of modeled cases of human trichinellosis. The same model suggests that wild boars have a 4100 times higher prevalence of *Trichinella* infection than pigs raised in non-controlled farming conditions [5].

Nevertheless, the prevalence found in wild boars in Europe may be characterized as low, as it has been found less than 1% in all cases. In some of the most recent, wide-range epizootiological surveys in Europe where AD methods were implemented, hundreds or thousands of wild boars were examined (in most cases these surveys were analysis of national authorities’ records); the prevalence of infection was 0.1% in Portugal [48], 0.17% in Croatia [49], 0.04% in Slovakia [50], 0% in Denmark [51], and 0.51% in Poland [52]. In a survey similar to the present study, conducted in Italy, diaphragm muscle samples of 100 wild boars hunted in two hunting seasons were examined, and none (0%) was found positive to *Trichinella* larvae [53]. Despite the generally low prevalence of infection, wild boar meat is to date the second, after pork, the most frequent source of human trichinellosis [54], presumably because it is the most popular game meat in most parts of Europe.

Interestingly, larvae of *T. britovi*, i.e., the only species identified in Greece so far in free-ranging domestic pigs and in humans [14,15], and presumably, the dominant species in the area, show significant differences in abundance and life span in pig muscles, compared to *T. spiralis* [55]. It was shown that 2 months post infection (p.i.), there is a remarkably lower number of *T. britovi* LPG in pig meat, i.e., 70 times lower than *T. spiralis*, and that these larvae had a shorter life span, as only a few survived for 6 months (none was detected 12 months p.i.), while *T. spiralis* were alive at least 2 years p.i. [55]. This short life span most probably results in a minimum accumulation of larvae in the muscles of wild boars and could explain the absence of larvae in the examined animals in Greece. In contrast to the short life span of larvae, antibodies can be detected for longer time periods (at least two years for both *T. britovi* and *T. spiralis*) and thus serosurveys investigating *Trichinella* infection in wild boars usually result in higher prevalences [55]. For example, in a serosurvey for *Trichinella* antibodies in wild boars in Greece, the seropositivity was 6.4% [16]. Similarly, antibodies were detected in 32 out of the 1462 wild boars in Italy, while in only one of them (1/1462), *T. britovi* larvae were found by AD [56].

*Alaria alata* mesocercariae can be found by the AD method applied obligatorily according to the current European regulations to all wild boars entering commercial circuits. As a result of the systematic *Trichinella* inspection, *A. alata* has been detected as an incidental finding in many European countries [6,57]. However, the samples for *Trichinella* inspection have to be free from fat, while *Alaria* shows a particular affiliation to adipose tissue [6]. Furthermore, it has been shown that apart from the diaphragm, the “cheek”, i.e., the various tissues included in the caudoventral region of the head (muscle, connective, fat, glandular, and lymphatic tissues) [28], as well as the tongue, muscle around the larynx, and intercostal muscles [58], are suitable spots for *Alaria* detection. It is thus reasonable to assume that the prevalence of *Alaria* found in the obligatory meat inspection for *Trichinella* in slaughterhouses is significantly underestimated. In some of the most recent surveys, conducted especially for the detection of *Alaria* mesocercariae, thus implementing the most sensitive direct method of detection, i.e., AMT [59], the prevalence found in European countries was 44.3% in northeastern Poland [26], 43.9% in Latvia [58], 11.5% in Germany [60], 10.3% in Serbia [61], 6.8% in the Czech Republic [62], 6% in Austria [63], 4.2% in Poland [64], 1.6% in Hungary [65], and 1% in northern regions of Italy [53]. In the most recent survey in Germany, the prevalence of infection in wild boar meat was 28.3%, i.e., the highest ever recorded in the country [66]. Similarly, Portier et al. [57] observed a clear and steady increase of *A. alata* detection in wild boar meat in the eastern areas of France (a rise in prevalence from 1.5% in 2007 to 6.6% in 2011), suggesting that this should be considered a true emergence of the parasite.

While wetlands are significant for the frequency of *Alaria* in wild boar meat, as the life cycle of the parasite includes two water-dependent organisms (water snails and amphibians), elevation also seems to play a role [57,58]. The average elevation of Greece is 247 m, however, most of the samples were collected from areas with an elevation of more than 300 m and this may have affected the results. Even though some of the examined wild boars were hunted close to wetlands, no infection with *Alaria* was detected in any of these samples. Finally, there is evidence that *A. alata* prevalence in wild boards is higher in the summer season, which is attributed to increased activity of the second intermediate host and to an adapted wild boar diet [58]. In the present study, most of the samples were collected during the winter months, i.e., the wild boar hunting season in Greece, which is generally between mid-September and mid-January. Mesocercariae of *A. alata* have never been found in Greece in any animal species. However, the parasite (eggs or adult trematodes) has been found in some species of final hosts in the country, e.g., in wolves (0.7%) [19], foxes (1.6%), jackals (20%) [17], wildcats (17.4%) [20], and dogs (2.5%) [18].

Sensitive and practical methods of *Alaria* mesocercariae detection are necessary for monitoring the parasite’s geographic distribution, prevalence, and range of paratenic hosts, in order to assess the parasite’s epizootiological trends and any risk for human infection. In this context, it has been repeatedly shown that even though the parasite may be found by the compression method and by AD, the most effective is the AMT, probably because mesocercariae are sensitive to the AD procedure and many are destroyed [28,58]. In a recent comparative study, Strokowska et al. [11] showed that of the 43 mesocercariae positive samples found by AMT, only 20 were positive by the magnetic stirrer AD method, and 25 by AD using Pancreatin^®^ bile and pancreatic enzymes, while the less sensitive method was compression. Even though AMT is the most effective method for mesocercariae detection, a combination of AD and AMT would reveal more *Alaria* positive meat samples than any one of these methods alone [58]. In the present study, each animal was examined by both methods, i.e., DP sample by AD and the MT by AMT, while additionally, compression method was applied to both samples, in an effort to increase the overall sensitivity of inspection. The negative result of these classical methods was confirmed by the very sensitive, newly developed real-time PCR.

Molecular diagnosis and identification of *A. alata* are based on previously developed conventional PCR [25], which uses primers targeting the large subunit ribosomal RNA gene. Furthermore, a matrix-assisted laser desorption/ionization time-of-flight mass spectrometry (MALDI-TOF) method has been recently tested for the identification of *Alaria* mesocercariae isolated from various hosts, with promising results [67]. In the present study, the previously designed primers [25] were modified to accommodate for real-time PCR conditions, and an LNA modified TaqMan probe was further designed for the specific detection and quantification of *Alaria* spp. genomic DNA, thus creating a tool for the confirmation of *Alaria* spp. infection in suspected samples. The developed methodology proved to be specific and in complete agreement with AMT. The developed real-time PCR showed 81.5% amplification efficiency, and this can be attributed to the large size of the amplicon (309 bp). However, it was proved to be sensitive (0.12 larvae per 50 g) and was able to test positive, animals with parasitic load from 1 up to 76 mesocercariae per 50 g of meat tissue. Importantly, the real-time PCR format exhibits minimum laboratory contamination risk and minimizes the chances of false-positive results, compared to gel-based assays. This assay is the first real-time *A. alata* detection method aiming at rapid diagnosis in clinical specimens. This method could be applied as a complementary to AMT diagnostic tool in future surveillance programs.

## 5. Conclusions

Trichinellosis is one of the most significant FBPD worldwide. Even though the modern systems of pig farming have diminished the infection in pig meat, game meat remains an important source of human infection. The increase of game meat consumption evidenced in recent years [10] renders the continuous vigilance and monitoring of this infection, at the level of meat inspection and at the level of surveillance surveys, an imperative. On the other hand, *A. alata*, as an agent of a potentially emerging disease and a possible threat to human health, should also be monitored in a more systematic way. It is thus important to increase awareness about this FBPD in the scientific community, food industry, and general public.

Toward this end, new, effective, and sensitive methods of detection that may be applied at the meat inspection level, as well as at the laboratory level, are needed. Indeed, the WHO supports the development and use of standardized methodologies for the assessment of FBD diseases in different countries, as a tool for reliable monitoring and effective, harmonized food safety policy [1]. Hopefully, the results of the present study contribute toward this end by describing a sensitive and specific real-time PCR that may be applied in various investigation settings of alariosis.

## Figures and Tables

**Table 1:** Primers, TaqMan probe, and amplification conditions used in the developed assay.

Assay/Amplicon Length	Primers and Probes	Sequence 5′→3′	Tm (°C)	μΜ	Cycling Conditions
Real-time PCR/309 bp	AlarDME1F2	CGCTTAGCTGCGGGTTCCTG	67.1	0.3	94 °C for 15′ 45 cycles: (a) 94 °C for 30″ (b) 59 °C for 45″ (c) plate read
	AlarDME1R2	CCAACGGCACATAAGCAAATACCTCG	67.4	0.3	
	AlAlprobe	FAM/TCT+T+A+CTGCTGTAGT+C+A+AAC/IBFQ	69	0.2	

+: Locked nucleic acid (LNA), to increase structural stability and hybridization temperature. FAM: 6-carboxyfluorescein; IBFQ: Iowa Black^®^ fluorescence quencher.

## Data Availability

All data produced in this study are provided herein.
